# Ascorbic acid partly antagonizes resveratrol mediated heme oxygenase-1 but not paraoxonase-1 induction in cultured hepatocytes - role of the redox-regulated transcription factor Nrf2

**DOI:** 10.1186/1472-6882-11-1

**Published:** 2011-01-03

**Authors:** Anika E Wagner, Christine Boesch-Saadatmandi, Dorothea Breckwoldt, Charlotte Schrader, Constance Schmelzer, Frank Döring, Koji Hashida, Osamu Hori, Seiichi Matsugo, Gerald Rimbach

**Affiliations:** 1 Institute of Human Nutrition and Food Science, Christian-Albrechts-University of Kiel, Hermann-Rodewald-Str. 6, 24118 Kiel, Germany; 2 Department of Neuroanatomy, Graduate School of Medical Sciences, Kanazawa University, Kakuma, Kanazawa, 920-1192, Japan; 3 College of Science and Engineering, School of Natural System, Kanazawa University, Kakuma, Kanazawa, 920-1192, Japan

## Abstract

**Background:**

Both resveratrol and vitamin C (ascorbic acid) are frequently used in complementary and alternative medicine. However, little is known about the underlying mechanisms for potential health benefits of resveratrol and its interactions with ascorbic acid.

**Methods:**

The antioxidant enzymes heme oxygenase-1 and paraoxonase-1 were analysed for their mRNA and protein levels in HUH7 liver cells treated with 10 and 25 μmol/l resveratrol in the absence and presence of 100 and 1000 μmol/l ascorbic acid. Additionally the transactivation of the transcription factor Nrf2 and paraoxonase-1 were determined by reporter gene assays.

**Results:**

Here, we demonstrate that resveratrol induces the antioxidant enzymes heme oxygenase-1 and paraoxonase-1 in cultured hepatocytes. Heme oxygenase-1 induction by resveratrol was accompanied by an increase in Nrf2 transactivation. Resveratrol mediated Nrf2 transactivation as well as heme oxygenase-1 induction were partly antagonized by 1000 μmol/l ascorbic acid.

**Conclusions:**

Unlike heme oxygenase-1 (which is highly regulated by Nrf2) paraoxonase-1 (which exhibits fewer ARE/Nrf2 binding sites in its promoter) induction by resveratrol was not counteracted by ascorbic acid. Addition of resveratrol to the cell culture medium produced relatively low levels of hydrogen peroxide which may be a positive hormetic redox-signal for Nrf2 dependent gene expression thereby driving heme oxygenase-1 induction. However, high concentrations of ascorbic acid manifold increased hydrogen peroxide production in the cell culture medium which may be a stress signal thereby disrupting the Nrf2 signalling pathway.

## Background

Resveratrol (3,4',5-trihydroxy-trans-stilbene) is a secondary plant metabolite which is highly abundant in red grape skin and red wine [1]. Other dietary sources of resveratrol comprise berries, and peanuts [2,3]. Although controversially discussed it has been recently shown that resveratrol may increase life span in model organisms such as Caenorhabditis elegans and Drosophila melanogaster [4-6]. The underlying mechanisms by which resveratrol may mediate beneficial effects have yet not been fully understood and may be partly related to its ability to induce phase II and antioxidant enzymes including heme oxygenase-1 and paraoxonase-1.

Heme oxygenase-1 (HO-1), is an inducible enzyme that catalyzes the rate-limiting step in the oxidative degradation of cellular heme that liberates iron, carbon monoxide (CO), and biliverdin. HO-1 exhibits antioxidant, anti-inflammatory and other cytoprotective functions [7].

Paraoxonase-1 (PON1) is a HDL associated serum enzyme which is mainly synthesized in the liver. PON1 may mediate anti-atherogenic properties by protecting low density lipoprotein (LDL) from oxidation [8]. PON1 deficient mice are highly susceptible towards atherosclerosis [9] whereas paraoxonase overexpression represses atherogenesis and promotes atherosclerotic plaque stability in mice [10].

Nuclear factor erythroid 2-related factor 2 (Nrf2) is a transcription factor that positively regulates the basal and inducible expression of a large battery of cytoprotective genes [11-13]. Following induction, Nrf2 is dissociated from the Keap1-Nrf2 complex, translocates to the nucleus where it binds to the antioxidant response element (ARE) to increase the expression of cytoprotective genes including HO-1.

Vitamin C (ascorbic acid) is an essential micronutrient in primates which exhibits some of its biological activity due to its free radical scavenging activity. Ascorbate is a water-soluble chain-breaking radical scavenger and recycles plasma membrane α-tocopherol via the reduction of the α-tocopheroxyl radical [14].

Beside antioxidant also prooxidant activities of ascorbic acid have been described [15,16]. Ascorbic acid promotes the Fenton reaction in vitro and may accelerate non enzymatic lipid peroxidation in various tissues [17]. Furthermore, pharmacological doses of ascorbate produced sustained ascorbate radical and hydrogen peroxide formation in mice [18]. Both resveratrol and ascorbic acid are frequently used in complementary and alternative medicine [19-22]. Little is known about interactions between resveratrol and ascorbic acid regarding the expression of genes encoding for antioxidant enzymes. Therefore in this study we have investigated if ascorbic acid affect resveratrol mediated induction of HO-1 and PON-1 gene expression. We have selected the two genes since they are centrally involved in cellular antioxidant defence mechanisms. Furthermore, both genes significantly differ in their number of ARE binding sites in their promoter region. ARE is an important binding motif for the redox regulated transcription factor Nrf2 which partly controls phase II and antioxidant gene expression.

## Methods

### In silico Analysis

For promoter analysis, sequences of HO-1 and PON1 genes were uploaded to *MatInspector *Software http://www.genomatix.de in order to identify putative binding sites for Nrf2. The respective promoter sequences were obtained from Ensembl genome browser http://www.ensembl.org and were adjusted to 2000 bp upstream and 600 bp downstream of transcriptional start site.

### Cell Culture

HUH7 human hepatoma cells (obtained from the Institute of Applied Cell Culture, Munich, Germany), were cultured in high glucose (4 g/l) Dulbecco's modified Eagle's medium supplemented with 10% (v/v) foetal bovine serum, 4 mmol/l L-glutamine, 100 U/ml penicillin and 100 μg/ml streptomycin (all PAA, Coelbe, Germany) and grown in a humidified incubator at 37°C and 5% CO_2_. HUH7/PON1 cells were cultivated in high glucose (4 g/l) Dulbecco's modified Eagle's medium with 10% heat inactivated foetal bovine serum, 100 U/ml penicillin, 100 μg/ml streptomycin and 100 μg/ml G418 (all from PAA, Coelbe, Germany). For cell culture studies stock solutions were prepared as following: 100 mmol/l resveratrol (Sigma, Deisenhofen, Germany) dissolved in DMSO, and stored at -80°C until further use; 1 mol/l ascorbic acid (Carl Roth, Karlsruhe, Germany) was freshly prepared in PBS and adjusted to pH 7. For all cell culture assays vehicle controls have been performed and did not affect any of the parameters measured.

### Cytotoxicity

The neutral red assay [23,24] was used to determine the cell viability after incubation with the different test compounds. HUH7 cells were seeded at a density of 0.2 × 10^6 ^cells/well (for HUH7) or 0.15 × 10^6 ^cells/well (for HUH7/PON1) for 24 h and treated with 5-100 μmol/l resveratrol and 10-1000 μmol/l ascorbic acid, respectively. HUH7 cells were incubated with test compounds for 24 h, HUH7/PON1 cells for 48 h. In brief, the culture medium containing the test substances was replaced with fresh serum-containing medium including 60 μg/ml of Neutral Red (Carl Roth, Karlsruhe, Germany). After incubation for 1.5 h the medium was removed and the cells were extracted using a solution comprising 50:49:1 (v/v/v) ethanol, water and glacial acetic acid. The absorbance was measured in a plate reader (Labsystems, Helsinki, Finland) at 540 nm.

### RNA isolation and real time PCR

HUH7 cells were seeded at a density of 1.0 × 10^6 ^cells/well in a 6 well plate for 24 h. Subsequently, cells were treated with resveratrol (10 μmol/l) alone and in combination with 100 μmol/l and 1000 μmol/l ascorbic acid for 6 h, respectively. RNA was isolated using TRIsure (Bioline, Luckenwalde, Germany) according to manufacturer's instructions. Primers for human HO-1 gene (forward 5'CCAGGCAGAGAATGCTGAGT 3'; reverse 5' GTAGACAGGGGCGAAGACTG3') were designed by Primer3 software and ordered at MWG Biotech/Eurofins, Ebersberg, Germany. Real time PCR was performed with a SensiMix one step kit (Quantace, Berlin, Germany).

### Stable transfection and luciferase reporter gene analysis for PON1

PON1 induction capacity of resveratrol in the presence of ascorbic acid was evaluated in cultured hepatocytes. HUH7 liver hepatoma cells of human origin had been stably transfected with a reporter plasmid containing 1009 bp [-1013, -4] of the PON1 gene 5'-region cloned into the firefly luciferase reporter vector pGL3 basic (Promega) as described previously [25]. Stable clones originated from X. Coumoul/R. Barouki, INSERM, France. HUH7/PON1 cells were seeded at an initial density of 150,000 cells per well (24 well plate) and incubated with 25 μmol/l resveratrol (Sigma, Deisenhofen, Germany) in the absence or presence of ascorbic acid (100 and 1000 μmol/l) for 48 h as recently described [26]. Then, the cells were washed with PBS, lysed and subjected to luciferase activity measurement (Luciferase assay system; Promega, Madison, WI, USA) by luminescence reading (Infinite 200 reader; Tecan, Crailsheim, Germany) and normalized to total cell protein (BCA Assay, Pierce, Illinois, USA).

### Transient transfection and luciferase reporter gene analysis for Nrf2

HUH7 cells were seeded in a 24-well-plate at a density of 0.1 × 10^6 ^cells for 24 h. Then cells were transfected with pARE_GIGPx_Luc (kindly provided by A. Banning/R. Brigelius-Flohé, DIFE, Potsdam, Germany), a luciferase reporter gene under the control of the ARE region found in the promoter of the human GIGPx, as described by Banning et al. 2005 [27]. The renilla reporter gene phRLTK (Promega, Mannheim, Germany) was applied for normalization. Both plasmids were transfected by using FuGene6 (Roche, Penzberg, Germany) according to manufacturer's protocol. 24 h post transfection medium was removed and the cells were simultaneously treated with 10 μmol/l resveratrol and 100 and 1000 μmol/l ascorbic acid for further 24 h. Following lysis with passive lysis buffer (Promega, Mannheim, Germany) firefly and renilla luciferase activity was determined using the Dual Luciferase Assay (Promega, Mannheim, Germany) according to manufacturer's description. Firefly luciferase values were normalized by renilla luciferase values. The experimental data represent the mean of three independent experiments performed in duplicate.

### Western Blotting

For HO-1 and PON1 detection cells were treated with the test compounds for 24 h. Subsequently, cells were washed with ice-cold PBS, scraped off, centrifuged and the remaining cell pellet was stored at -80°C. For Western Blotting, the cell pellet was lysed in RIPA buffer [50 mmol/l Tris-HCl, 150 mmol/l NaCl, 0.5% (w/v) sodium deoxycholate, 0.1% (w/v) sodium dodecyl sulphate (SDS), 1% (w/v) Nonidet-P40, 2 mmol/l EDTA, 1 mmol/l dithiothreitol (DTT), protease inhibitor cocktail (Sigma, Deisenhofen, Germany)] and stored until further analysis. Protein concentrations were determined with the BCA assay (Pierce, Illinois, USA) according to manufacturer's instructions. 40 μg protein of each sample were mixed with loading buffer, denatured at 95°C for 5 min and separated on a 12% SDS PAGE. Subsequently the samples were transferred onto a PVDF membrane and blocked with 5% (w/v) skim milk dissolved in TBS+0.05% (v/v) Tween-20 (TBST) for at least 1 h and probed with HO-1 (Stressgen, Michigan, USA; 1:1000) or PON1 (Abcam, Cambridge, UK; 1:1000) at 4°C over night. Following, the membranes were incubated with a secondary antibody (1:4000 anti-rabbit) for 45 min and the bands were visualized by using ECL reagent in a ChemiDoc XRS system (both BioRad, Munich, Germany).

### Fox Assay

Fox assay was carried out according to Long and coworkers [28]. Tests were performed in DMEM high glucose medium supplemented with 10% fetal bovine serum and 100 U/ml of penicillin and 100 μg/ml streptomycin. DMEM was supplemented with increasing concentrations of resveratrol (1, 5, 10, 25, 50 μmol/l) in combination with 100 and 1000 μmol/l ascorbic acid (adjusted to pH 7), respectively. A standard curve was prepared using H_2_O_2 _(0, 2.2, 8.8, 22, 88 μmol/l). The samples were mixed with FOX reagent and incubated at room temperature for 30 min. Following centrifugation at 15,000 × g for 4 min the absorbance was read at 560 nm.

### Statistical Analysis

Statistical analysis was conducted using SPSS software Version 15.0 (Munich, Germany). Data were analysed for normality of distribution (Kolmogorov-Smirnov or Shapiro-Wilk-test). In case of not normally distributed data the non-parametric Mann-Whitney-U test was applied. One-way analysis of variance (ANOVA) with a Dunnet's (homogeneous variances) or Games-Howell (heterogenous variances) post hoc test was performed. Data are expressed as mean ± SEM. Significance was accepted at p < 0.05.

## Results

### 1) In silico analysis

**Figure 1:**
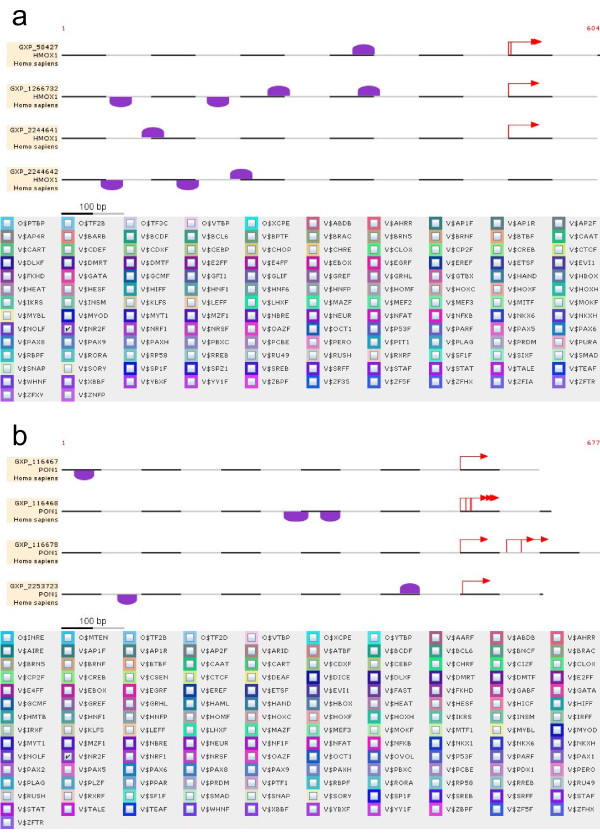
**Nrf2 binding sites in alternative promoter sequences of the HO-1 and PON1 gene**. Nrf2 binding sites are depicted in alternative promoter sequences of (a) HO-1 (GXP_58427, GXP_1266732, GXP_2244641, GXP_2244642) and (b) PON1 (GXP_116467, GXP_116468, GXP_116678, GXP_2253723) relative to transcription start site (red arrow).

To identify binding sites for Nrf2 in promoter regions of HO-1 and PON1, the respective promoter sequences were uploaded to *MatInspector*. As shown in figures 1a and 1b, four alternative promoter sequences have been identified for both HO-1 and PON1 genes. Importantly, binding sites (in total eight) for Nrf2 have been identified in all promoter sequences of HO-1 (Figure 1a). However, for the PON1 gene, only three promoter sequences have been identified comprising in total five Nrf2 binding sites (Figure 1b).

### 2) Cell culture experiments

**Figure 2:**
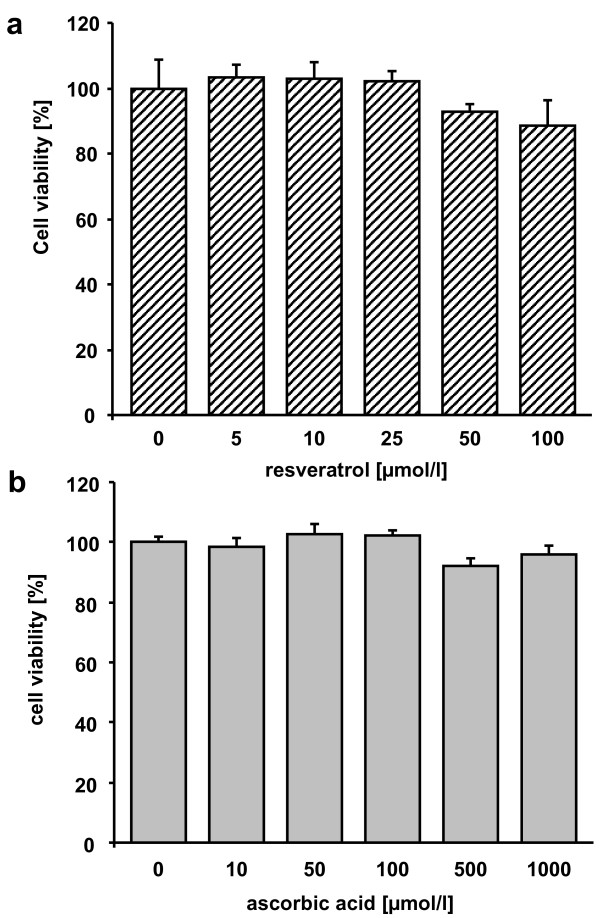
**Cytotoxicity of resveratrol and ascorbic acid**. Effects of resveratrol (a) and ascorbic acid (b) on cell viability in HUH7 cells. Data are mean ± SEM of at least 2 experiments performed in triplicate.

Resveratrol was not cytotoxic up to a concentration of 25 μmol/l. At 50 μmol/l resveratrol a slight 10% decrease in cell viability was observed (Figure 2a). Ascorbic acid (Figure 2b) did not exhibit any cytotoxicity up to a concentration of 1000 μmol/l each.

**Figure 3:**
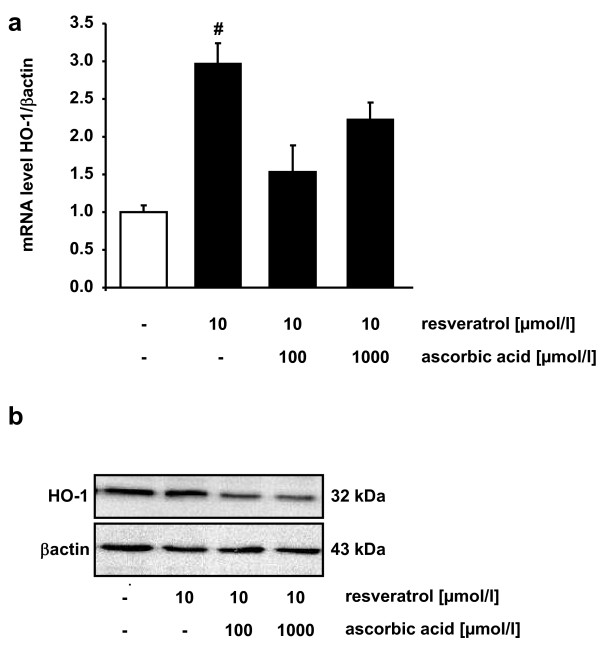
**Effects of resveratrol and ascorbic acid on HO-1 mRNA and protein levels**. mRNA (a) and protein levels (b) of HO-1 in HUH7 cells in response to resveratrol supplementation in the absence and presence of 100 and 1000 μmol/l ascorbic acid. Data are mean ± SEM of at least 3 experiments performed in triplicate. # indicates significant difference between control and 10 μmol/l resveratrol p < 0.05, Mann-Whitney-U-Test.

Incubation of HUH7 cells with 10 μmol/l resveratrol resulted in a significant increase in HO1 mRNA (Figure 3a) and protein levels (Figure 3b). Coincubation of resvertraol with ascorbic acid partly counteracted resveratrol mediated HO-1 induction.

**Figure 4:**
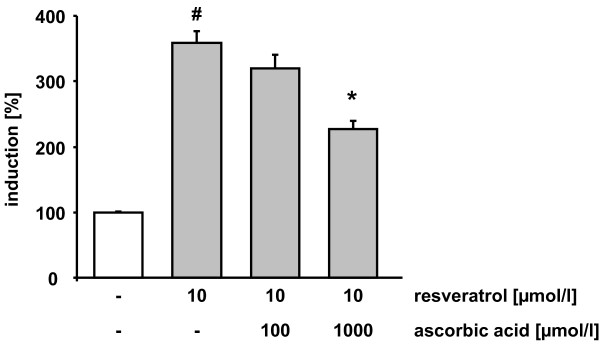
**Effects of resveratrol and ascorbic acid on Nrf2 transactivation**. HUH7 cells were supplemented with resveratrol in the absence and presence of 100 and 1000 μmol/l ascorbic acid. Data are mean ± SEM of at least 3 experiments performed in triplicate. # indicates significant difference between control and 10 μmol/l resveratrol p < 0.05, Mann-Whitney-U-Test; * indicates significant differences compared to 10 μmol/l resveratrol p < 0.05, Mann-Whitney-U-Test.

HO-1 gene expression is highly regulated by the transcription factor Nrf2. Therefore we determined Nrf2 transactivation in the absence and presence of resveratrol and ascorbic acid. Resveratrol significantly enhanced Nrf2 transactivation (3.5 fold increase as compared to untreated cells). Coincubation of resveratrol with 1000 μmol/l ascorbic acid antagonized resveratrol mediated Nrf2 induction (Figure 4).

**Figure 5:**
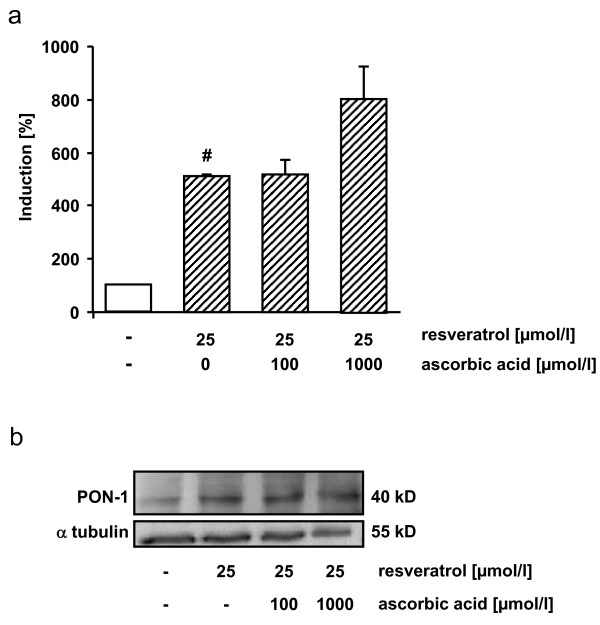
**Effects of resveratrol and ascorbic acid on PON1 transactivation and PON1 protein levels**. PON1 transactivation in HUH7/PON1 cells supplemented with resveratrol in the absence and presence of 100 and 1000 μmol/l ascorbic acid (a). Protein levels of PON1 in HUH7 cells in response to resveratrol supplementation in the absence and presence of 100 and 1000 μmol/l ascorbic acid (b). Data are mean ± SEM of at least 2 experiments performed in triplicate. # indicates significant difference between control and 25 μmol/l resveratrol p < 0.05, Mann-Whitney-U-Test;

Incubation of HUH7 cells stably transfected with PON1 with resveratrol significantly increased PON1 transactivation (Figure 5a). Ascorbic acid concentration of 100 μmol/l did not affect PON1 transactivation, whereas 1000 μmol/l ascorbic acid slightly enhanced PON1 transactivation. Furthermore resveratrol induced PON1 protein levels in HUH7 cells. The induction of PON1 due to resveratrol was not counteracted by ascorbic acid (Figure 5b)

### 3) Fox-Assay

**Figure 6:**
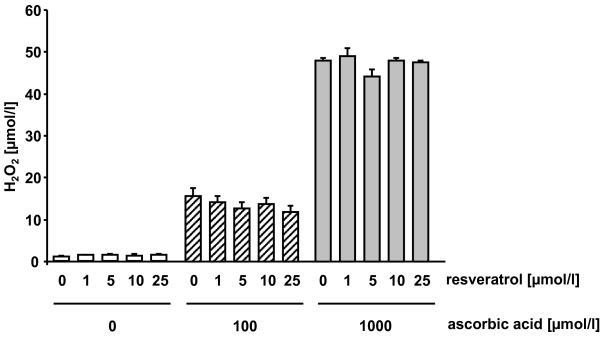
**Production of hydrogen peroxide by resveratrol and ascorbic acid**. Production of hydrogen peroxide in DMEM medium supplemented with resveratrol in the absence and presence of ascorbic acid. Data are mean ± SEM of at least 2 experiments performed in triplicate.

As shown in figure 6 resveratrol per se produced relatively low amounts (1.3-1.6 μmol/l) of hydrogen peroxide in the DMEM cell culture medium as determined with the Fox assay. The addition of 100 μmol/l and 1000 μmol/l ascorbic acid resulted in a dose-dependent increase in hydrogen peroxide formation. At 100 μmol/l ascorbic acid ~15 μmol/l hydrogen peroxide and at 1000 μmol/l ascorbic acid almost 50 μmol/l of hydrogen peroxide were generated.

## Discussion

In the present study we demonstrate that ascorbic acid partly antagonizes resveratrol mediated HO-1 but not PON1 induction in cultured human hepatocytes. Resveratrol is known to induce HO-1 in cell culture as well as in the liver of laboratory rats [29-31]. As far as the concentrations of the test components are concerned administered ascorbic acid concentrations, as used in our cell culture experiments, are by in large in the physiological range [32]. However, resveratrol concentrations used in this study in order to induce HO-1 and PON1 are higher than plasma values reported in humans [33]. Interestingly, under the conditions investigated, ascorbic acid counteracted the induction of HO-1 by resveratrol. Ascorbic acid is a water soluble cytosolic antioxidant. Thus the decrease in HO-1 mRNA and protein levels due to ascorbic acid may be mediated by effects which occur in the cytosol.

Although ascorbic acid moderately antagonized resveratrol mediated HO-1 induction we have found no antagonistic interaction between resveratrol and ascorbic acid in terms of PON1 transactivation and its protein levels. While the HO-1 promoter region contains many Nrf2/ARE binding sites, PON1 is regulated to a lower extend by Nrf2. Therefore, we suggest an important role of the transcription factor Nrf2 in the antagonism between resveratrol and ascorbic acid regarding HO-1 gene expression.

Since the addition of 1000 μmol/l ascorbic acid to the DMEM medium resulted in the production of significant levels of hydrogen peroxide as previously described [34] it may be possible that hydrogen peroxide directly disrupted the Nrf2 signalling pathway leading to a decreased HO-1 gene expression. Thus under conditions investigated ascorbic acid exhibited prooxidant activity which in turn may have attenuated resveratrol induced HO-1 induction. However, it needs to be taken into account that the FOX assay is not specific for hydroperoxides; also cyclic peroxides and serial-cyclic peroxides may give positive FOX response [35].

**Figure 7:**
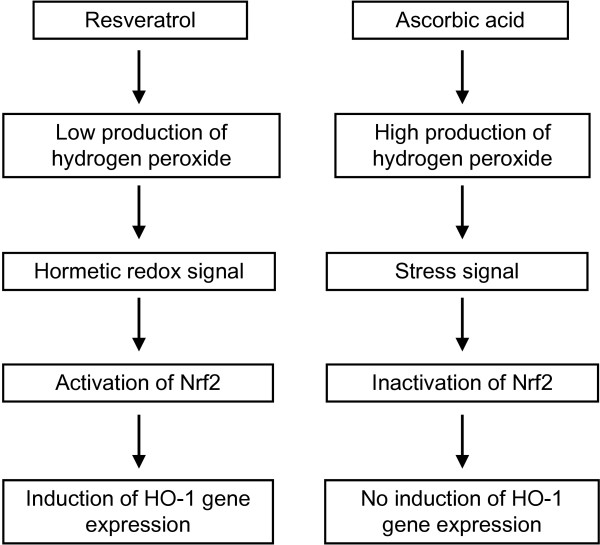
**Potential molecular mechanisms by which resveratrol and ascorbic acid may affect Nrf2 signalling**.

In this context it has been recently shown that free radicals deriving from cigarette smoke increase protein carbonyl formation of the Nrf2/Keap1 complex. This in turn leads to modifications of sulfhydryl groups of Nrf2/Keap1 which may impair the dissociation of the Nrf2/Keap1 complex and consequently repressing the nuclear translocation of Nrf2. The modified Nrf2 may then undergo proteasomal degradation [36]. Interestingly addition of resveratrol to the cell culture medium produced in our study low levels of hydrogen peroxide which may be a positive "hormetic redox-signal" for Nrf2 dependent gene expression thereby driving HO-1 induction. Contrary ascorbic acid is inducing the production of high levels of hydrogen peroxide which may be considered as a "cellular stress signal" that interferes with the Nrf2 signal transduction cascade thereby antagonizing HO-1 gene expression (see Figure 7). Interestingly Ristow and coworkers have recently demonstrated that ascorbic acid may interfere with physical exercise related induction of the Nrf2 target genes superoxide dismutase and glutathione peroxidase in humans [37].

The generation of reactive oxygen species by ascorbic acid, as shown in this cell culture study, my not be always detrimental. It has been shown that pharmacological concentrations of ascorbic acid increased the production of hydrogen peroxide in pancreatic cancer cells which in turn resulted in the induction of caspase-independent cell death and autophagy [38]. Furthermore, it should be taken into account that disruption of Nrf2 activity by ascorbic acid may result in a GSH depletion which could restore the sensitivity of cancer cells towards anticancer drugs such as imatinib [39].

## Conclusions

Overall the current study has some limitations: First, our findings are based on cell culture studies. Thus further studies in laboratory rodents and in humans are needed to investigate interactions between resveratrol and ascorbic acid on HO-1 and PON1 status. Second, the concentration of resveratrol as used here in cultured cells to induce HO-1 and PON1 are higher than those physiologically achievable. Finally we have worked with purified test components only. It has yet not been investigated whether the observed interactions between reveratrol and ascorbic acid on HO-1 and PON1 status may also occur when resveratrol and ascorbic acid derive from a complex diet.

## Competing interests

The authors declare that they have no competing interests.

## Authors' contributions

AEW, CBS, DB, CS, CS, KH, OH performed experiments and analysed data. AEW, FD, SM and GR designed the study. AEW and GR drafted the manuscript. All authors read and approved the final manuscript.

## Pre-publication history

The pre-publication history for this paper can be accessed here:

http://www.biomedcentral.com/1472-6882/11/1/prepub
